# Midwifery and Diseases of Women

**Published:** 1891-11

**Authors:** C. H. Burford


### Selections.

### MIDWIFERY AND DISEASES OF WOMEN.

### ON THE CONTINUOUS IMPROVEMENTS IN METHOD IN CESARIAN SECTION.

FRAENKEL (ERNST)—*Deutsche med. Wochenschrift*, Nos. 33 and 34, gives a concise summary of the gradual evolution of technique, from the crude old operation to the perfected methods of the latest scheme. He insists also upon the simplification necessary in the operative details, in order to place the procedure within the working area of the general practitioner; and puts it upon the level of tracheotomy in croup, and herniotomy in strangulated gut — accomplishments which it is within the power of the majority to possess.

Up to the introduction of Porro's operation, a decade back, the mortality of the old method of Cesarian section was about ninety per cent. A notable epoch was made by Porro's plan, the success following which was much greater, whilst the great objection consisted in the extensive mutilation necessary. Feeling the firmer ground reached by Porro, Sänger published a few years afterwards the details of a well-considered plan, by which the success of Porro should be maintained, whilst the objectionable mutilation should be obviated. Sänger's *Restitutio in Integrum*, somewhat modified, is the operation of the present day, and Fraenkel claims it as an advance as great as that of Langenbeck's subperiosteal resections over the old method of dismemberment.

Fraenkel points out that Sänger's plan differs in two main features from the old operation, namely: in the asepsis to be maintained, and in the plan of suturing. To this latter the author attached the greatest possible importance; as, in his view, the success of the operation depended on the prevention of sanguineous or lochial discharge from entering the serous cavity, and in the union of the uterine wound throughout by first intention. The plan of suturing and the material for suturing were of equal importance. The former Sänger gave under three heads: a muscular suture series, which was sufficiently numerous and sufficiently strong to practically seal the uterine wound from leakage; the perforation of these sutures on the endometrial side, so as to miss the decidual tissue, thus avoiding later wound infection ; and finally, the closing of the serous visceral incision by a special row of sutures, any redundant serous membrane being cut away, and each serous edge infolded. The material used was silver wire.

Fraenkel shows at some length how and why the use of silver wire was gradually abandoned, how catgut was extensively tried, and finally how and why silk has been installed as the sole material for suturing. He records a case in which peritonitis spoilt an otherwise promising operation, it resulting from gaping in one or two spots of the uterine wound, where catgut had been used. Silk, also, is a stitch material within the reach of all, requiring no technical preparation for use. With the use of silk as suture, he shows further how it has not been found necessary to avoid puncturing the decidual membrane in suture introduction ; that it is not necessary to use a special row of sutures for the divided peritoneum, but that strong silk sutures, penetrating the serosa, muscle, and decidual membrane alike, may be used, and knotted independently on the serous aspect of the uterus.

A detailed account of a successful operation is given. The vagina was well cleansed with a three per cent. carbolic lotion. A median long abdominal incision was made, commencing four fingers' breadth over the umbilicus. The uterus was raised up, an elastic ligature placed loosely around the lower segment, and a twelve cm. long incision made into the uterine wall. This struck the placental site, the presenting part of which was detached, and the fetus rapidly delivered. The elastic ligature was now made tight, a sponge wrung out of sterilized salt solution placed in the uterine cavity, and the suturing of the uterine incision commenced. Sixteen strong silk sutures were inserted, perforating the peritoneum about one cm. from the wound edge and passing through serosa, muscle, and decidua successively. The sponge was removed, the sutures tied and knotted on the peritoneal side, and the last of a series of four hypodermic injections of ergotin, which had been given at short intervals during the suturing, was inserted. The elastic ligature was now removed, the uterus had well contracted but still some oozing occurred in three spots in the line of incision. An additional suture was inserted at each bleeding spot, and found sufficient to check further flow. The remainder of the operation was concluded in the usual manner.

[The late Carl Braun advocated and practised in the Vienna

Universitäts-Klinik, the plan of suturing the slit in the parietal peritoneum so as to include the slit in the visceral peritoneum also. In this way adhesion of the serous surfaces at the lines of incision occurred, the general peritoneal sac was cut off, and exit for any wound secretions took place through the lower angle of the parietal incision purposely stuffed with iodoform wick.]

Fraenkel concludes by discussing the principal elements of the operation at some length. The general scheme is to simplify the details as much as possible, so as to bring the operation easily within the skill and the resources of the general practitioner. The necessity for strict asepsis of hands and instruments, and dressings, and the "dry treatment" of the peritoneum is insisted on.

Three assistants are necessary: one to assist the operator, another to be responsible for the instruments, and the third to anesthetise. The preliminary hypodermic of atropine-morphine often given, is, in these cases, to be omitted, the former alkaloid interfering with the prompt contraction of the uterus, so essential to success. To ensure this latter, several hypodermics of ergotin are given, beginning directly after the extraction of the fetus. The lifting up of the uterus through the abdominal incision is desirable, partly to allow the easy passing of the elastic ligature, and partly to give space for the insertion of three or four provisional sutures in the parietes, to prevent uterine fluids from entering the serous cavity. The quicker the uterine suturing is conducted, the shorter is the time for full constriction by the elastic ligature, and the less the risk of uterine atony following its removal.

Not every case is suitable for the conservative operation. Where a marked pyrexia already exists, or the decidual membrane shows distinct signs of decomposition or disintegration, or if the operative interference has been unduly deferred, or efforts at delivery made by persons of indifferent cleanliness, then the propriety of the Porro as diminishing the risk of peritonitis is to be considered. In doubtful cases, Fraenkel advises Porro's operation as safer than the Kaiserschnitt. C. H. Burford.

—*Medical Chronicle.*

